# Principles, mechanisms and functions of entrainment in biological oscillators

**DOI:** 10.1098/rsfs.2021.0088

**Published:** 2022-04-15

**Authors:** Alba Jiménez, Ying Lu, Ashwini Jambhekar, Galit Lahav

**Affiliations:** ^1^ Department of Systems Biology, Blavatnik Institute at Harvard Medical School, Boston, MA 02115, USA; ^2^ Ludwig Center at Harvard, Boston, MA 02115, USA

**Keywords:** entrainment, biological oscillators, synchrony, phase response curve, Arnold tongue

## Abstract

Entrainment is a phenomenon in which two oscillators interact with each other, typically through physical or chemical means, to synchronize their oscillations. This phenomenon occurs in biology to coordinate processes from the molecular to organismal scale. Biological oscillators can be entrained within a single cell, between cells or to an external input. Using six illustrative examples of entrainable biological oscillators, we discuss the distinctions between entrainment and synchrony and explore features that contribute to a system's propensity to entrain. Entrainment can either enhance or reduce the heterogeneity of oscillations within a cell population, and we provide examples and mechanisms of each case. Finally, we discuss the known functions of entrainment and discuss potential functions from an evolutionary perspective.

## 1. Introduction

Oscillating systems can interact with each other in various ways. They can enhance or negate each other's effects (constructive and destructive interference, respectively) or synergize with each other to achieve amplitudes greater than the sum of the two systems (resonance). When two oscillating systems interact, one or both can experience an alteration in frequency to become phase-locked, meaning that the phase difference between the two oscillating systems remains constant in time and is robust to perturbations [1]. This situation is called entrainment.

**Figure 1.**
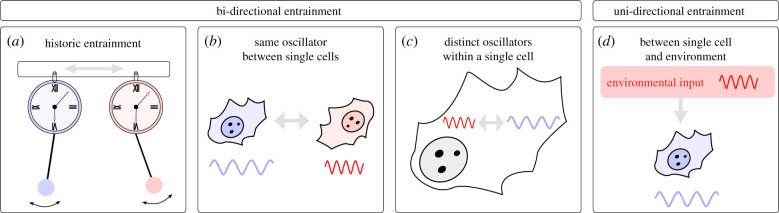
Entrainment types and their directionality. (*a*) Entrainment as originally described between two physically connected oscillating pendulums. (*b*) Entrainment of the same oscillator in two neighbouring single cells. (*c*) Entrainment of distinct oscillators within a single cell. (*d*) Entrainment of an oscillator within a single cell by an external periodic input.

Entrainment was originally described as two pendulum clocks coupled through a wooden structure [2] (figure 1*a*). Synchronization in this system was achieved via mechanical vibrations through the wooden coupling bar. Oscillations are also found in various biological systems and can operate at the molecular level (e.g. cardiac cell beating) or at the organismal level (e.g. sleep–wake cycles). Entrainment of these oscillations can occur through interactions between single cells, within a single cell or between a cell and its environment (figure 1*b*,*d*).

Biological oscillators can entrain in a variety of ways. Two biological oscillators in neighbouring cells can interact and influence each other through their extracellular environment (figure 1*b*). Entrainment between cells often occurs through secreted factors and therefore becomes apparent as cell density increases [3–6]. It allows coordination between cells in a tissue in order to perform a function: for example, cardiac cells synchronize their oscillations in order to provide a strong single voltage that leads to heart contraction [7,8]. Two biological clocks can also entrain *within* a single cell, as observed between the circadian and cell cycle oscillators (figure 1*c*). Entrainment of oscillators within a single cell allows for synchronizing the processes controlled by the two individual oscillators. Last, the frequency of a biological clock can entrain to an environmental periodic rhythm (figure 1*d*) that is itself unaffected by the biological oscillator. The most prevailing example of such unidirectional entrainment is the circadian clock, in which sleep–wake cycles entrain to light–dark cycles [9,10]. Entrainment between a cell and its environment allows organisms to keep their physiology in synchrony with their surrounding rhythms.

## 2. Key principles of entrainment

Entrainment depends on two basic conditions: (i) the coupling strength between the oscillator and external input and (ii) the similarity between the intrinsic frequencies of the internal oscillator and the external input in the absence of interaction [1]. Generally, a stronger coupling strength and closer intrinsic frequencies favour entrainment, though the exact requirement varies in different systems. The entrained state (or locked state) is represented by a rational number *p*/*q*; after *p* periods of the internal oscillator and *q* periods of the external oscillator, the system returns to the same state. As the coupling strength increases, phase locking becomes possible at a wider range of external periods (depicted by a broadening of Arnold tongues, see box 1), and the entrained mode is more robust against random fluctuations. Further increasing coupling strength may result in complex phenomena such as multi-stability, in which multiple entrainment modes coexist, and chaos. These are depicted by the overlap of different ‘tongues’. The transition between a robust locked state and a chaotic one has been observed in a classic example of periodically stimulated cardiac cells, in which a small variation of the period of the electrical stimuli caused a transition between normal and pathological behaviour of cardiac tissue (dysrhythmia) [13].

**Figure 2.**
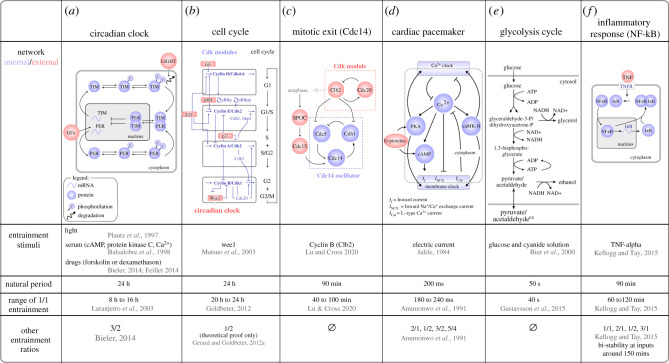
Six biological cases of entrainment. For each oscillator, the internal minimal network (blue) and external nodes (red) are portrayed, along with the stimuli used for entrainment and the observed entrainment ratios. (*a*) The fly circadian clock is regulated at the levels of transcription, protein stability and post-translational modifications [14]. It responds to light and GFs, but it can oscillate freely in the dark [9]. (*b*) The mammalian cell cycle network contains four coupled modules each centred around one cycle/Cdk complex which promotes progression or transition into the ordered succession of the cell cycle phases G1, S, G2 and M. The cell cycle components Wee1, p21 and cyclin E are transcriptionally regulated by the circadian clock [15]. (*c*) The Cdc14 network module is a negative feedback loop controlling cycles of nucleolar sequestration and release of Cdc14, which is essential for mitotic exit in budding yeast [16]. Each component of this loop (Cdc14, Cdc5, Cdh1) is coupled to the cell cycle. (*d*) Cardiomyocytes of the sinoatrial node (SAN) autonomously oscillate through action potentials that result from the opening and closing of sodium, calcium and potassium channels in their membrane, creating depolarization and repolarization oscillations [17,18]. (*e*) Glycolysis consists of the step-by-step breakdown of glucose and storage of the released Gibbs energy in the form of ATP. Oscillations correspond to changes in the concentration of glycolytic metabolites nicotinamide adenine dinucleotide plus hydrogen (NADH) and ATP. The molecular mechanism for oscillations is based on the speed of enzymatic reactions [12]. Sustained glycolytic oscillations require both glucose and cyanide to be present in the medium [19,20]. (*f*) The transcription factor NF-κB oscillates between the cytoplasm and nucleus in response to the inflammatory signal TNF-alpha [21,22]. TNF-alpha signalling induces the dissociation of the I*κ*B::NF-κB complex in the cytoplasm, allowing NF-κB to enter the nucleus and activate transcription of its inhibitor I*κ*B, which sequesters NF-κB in the cytoplasm [23].

Box 1. **The coupling and uncoupling between the oscillator and external input can be summarized in an ‘Arnold tongue’ plot. The Arnold tongue plot can be interpreted in the following way: with a fixed coupling strength (*y*-axis), if the intrinsic frequency of one oscillator traverses horizontally across the Arnold tongue plot, the coupled system will either stay not locked (case b) or be locked into distinct frequency modes featuring fixed *p*/*q* ratios (cases a and c). Phase locking is defined by measuring *ϕ*(*t*) and Δ*ϕ*(*t*), with *ϕ*(*t*) being the phase of an oscillator relative to the start of the cycle, expressed as a fraction of the period *ϕ*(*t*) ∈[0,2π], and Δ*ϕ*(*t*) being the difference in phase between two periodic signals at a given time Δ*ϕ*(*t*) = *ϕ*_oscillator1_(*t*) − *ϕ*_oscillator2_(*t*). When the phase difference Δ*ϕ*(*t*) between two signals is constant in time, the two signals are considered to be phase-locked. Traversing vertically over the plot (increasing coupling strength) illustrates how the coupled system becomes more robust against fluctuations (broadening of Arnold tongues) or can lead to multi-stability (case d) or other irregular dynamics such as chaos. ‘Tongues’ associated with high-order entrainment modes (5/4, 3/2, etc.) are usually smaller than that for the equal-frequency model (1/1) and therefore harder to observe experimentally (figure 2, *Other entrainment ratios*). The amplitude during entrainment remains unaltered as shown both theoretically [11] and experimentally [12].**

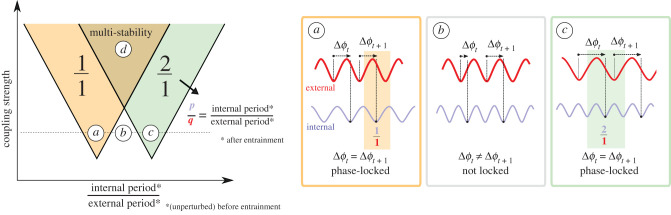

## 3. Examples of biological oscillators exhibiting entrainment

We will explore entrainment focusing on six biological examples of autonomous oscillators, which have been shown to entrain experimentally (figure 2). For each example, we present the simplest model that accounts for oscillatory behaviour, along with the node(s) receiving the stimuli for entrainment. Each example follows the basic principle of biochemical oscillators but differs in terms of its network architecture, the nature of the oscillations, their time-scale and the number of entrainment modes. All examples have a negative feedback loop within their core network (see blue networks in figure 2) with additional positive feedback loops providing robustness [24]. Other details of the networks vary with regard to the number of nodes, number of positive and negative interactions and number of points of coupling to external oscillators. In addition, the oscillating factors differ between the various systems. For example, in the circadian clock example, mRNA and protein levels oscillate [25], nuclear factor kappa B (NF-κB) and Cdc14 oscillate in their nuclear–cytoplasmic localization [23,26] and the glycolysis network oscillates in the products of enzymatic reactions [27]. The time-scale of oscillations also varies between these systems, with transcriptionally regulated systems exhibiting longer time-scales (hours for NF-κB and the circadian clock) and oscillators relying on enzymatic reactions operating on shorter time-scales (less than a minute for glycolytic oscillations) (figure 2, *Natural period*). In this review, we will not focus on systems that show irregular oscillations, such as bursting dynamics of calcium ions [28], nuclear translocation of Msn2 [29], insulin secretion by B-cells [30] or neuron spiking [31].

The six biological examples covered here have been extensively modelled using ordinary differential equations (ODEs) to describe their regulatory networks [21,32–37]. Dynamical systems tools, such as ODEs, phase portraits and bifurcation diagrams, are keys to understand how various systems differ in their requirements for initiating and sustaining oscillations [38]. For example, a model of the cell cycle [39] shows self-sustained oscillations only in the presence of growth factor (GF), thus defining GFs as a trigger between quiescence (non-oscillatory state) and proliferation (oscillatory state). Glycolytic oscillations require both glucose and cyanide to be sustained [19,20], with glucose alone leading to dampened oscillations but the addition of cyanide leading to sustained oscillations. GF and cyanide are thus Hopf parameters that are responsible for a Hopf bifurcation [20,36,39], meaning that they lead the system to transition from steady state (non-oscillatory) to a limit cycle (self-sustained oscillations). In most cases, oscillation triggers (Hopf parameters) also serve as entrainment stimuli. For example, entrainment of glycolytic oscillations by cyanide [20], or entrainment of NF-κB by tumour necrosis factor (TNF) [40], but that is not always the case, for example GFs only initiate but cannot entrain the cell cycle [39].

## 4. Distinguishing between entrainment and other mechanisms leading to synchrony

Synchrony is the empirical observation of two systems oscillating in phase, which can result from either entrainment or other mechanisms such as gating [41,42]. During entrainment, all phases of the follower oscillator must be affected by the leading oscillator—in other words, the oscillatory curve of the follower must be proportionately stretched out or compressed through all phases. By contrast, during gating, the leading oscillator defines windows of time in which different phases of the follower oscillator can occur. As opposed to entrainment, a gating mechanism follows these three principles: (i) arresting the lead oscillator at any constant level will arrest the follower oscillator; (ii) only 1 : 1 ratios will be observed; and (iii) the leading oscillator impacts only specific phases of the follower oscillator.

Distinguishing between gating and entrainment mechanisms has met with varying degrees of success. Strong evidence in favour of entrainment was obtained for the coordination between the cell cycle and Cdc14 nucleolar sequestration and release [43]. Blocking the cell cycle by maintaining cyclin B at constant physiological levels did not block Cdc14 oscillations, ruling out a gating mechanism. The mechanisms governing other synchronized systems, such as the synchronization between the cell cycle and circadian rhythm, have not reached consensus. Among the studies in favour of entrainment [15,33,44], Feillet *et al*. [44] reset the circadian clock using a glucocorticoid agonist and observed a variety of coupled states between the clock and the cell cycle (1 : 1, 1 : 2, 3 : 2), supporting an entrainment mechanism and aligned with computational studies [15]. Among the studies suggesting a gating mechanism [45–47], Laranjeiro *et al*. [45] manipulated light–dark cycles in zebrafish cells to vary the period of the circadian clock and observed an exclusive effect on the length of G1 with S/G2/M phases remaining relatively constant. As articulated above, impact over specific phases of the follower oscillator is characteristic of a gating mechanism.

Most studies in favour of entrainment between the circadian and cell cycle oscillators consider unidirectional entrainment with the circadian clock unidirectionally entraining the cell cycle (figure 1*d*). Circadian rhythms persisted in cells whose division was inhibited, initially suggesting unidirectional entrainment [48]. However, the possibility of bi-directional entrainment has not been ruled out [15]. It is plausible that altered cell cycle dependent changes in transcription or reduced protein concentrations after cell division may affect the circadian phase [49–51]. Future work using synthetic biology approaches to study isolated or minimally coupled oscillators could help elucidate both the mechanisms leading to synchrony in other systems (entrainment versus gating) and the directionality of entrainment (uni- versus bi-directionality).

## 5. Different biological oscillators vary in their propensity for entrainment

The study of entrainment can be greatly simplified by studying the response of an oscillator to a single pulse instead of to a periodic input. Such single perturbation is often shorter than the period of the oscillating system and can cause a shift in the original phase, either advancing or retarding the oscillations depending on its start time relative to the phase of the natural oscillator. A common way to capture this dependency is through phase response curves (PRCs) [52,53]. The features of a PRC, such as its magnitude (amplitude in the *y*-axis), zero points (intercept of the *x*-axis) and discontinuities (i.e. phase singularities), impact the propensity for entrainment [1] (box 2).

Box 2. **The inclination of a system to be entrained depends on its sensitivity to the perturbation and can be interpreted from the shape and properties of the phase response curves (PRCs). A PRC describes the magnitude of phase changes (also called phase shift) by plotting how much the oscillation is shifted in time (i.e. new phase *ϕ*_new_ minus unperturbed old phase *ϕ*_old_ on the *y*-axis) as a function of the phase at which it is received (*x*-axis).**

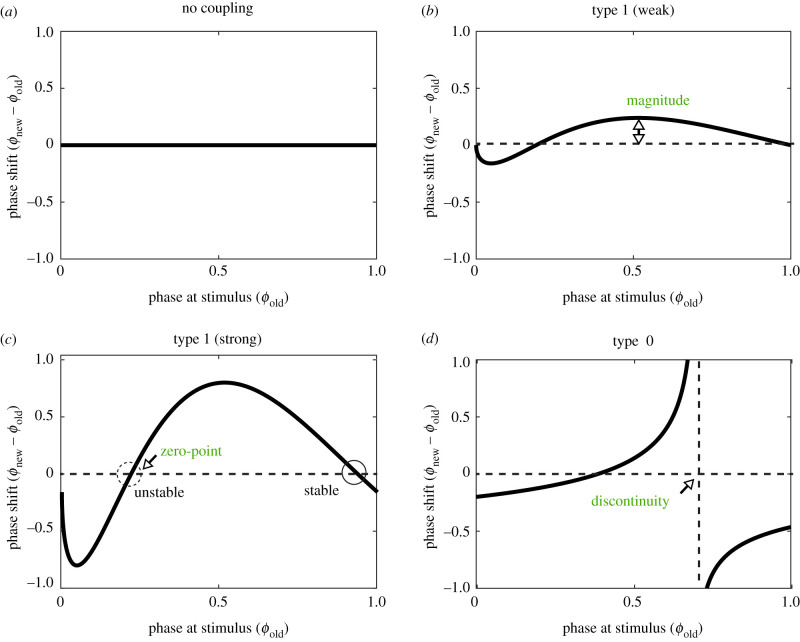

A system's PRCs can change by varying the amplitude or duration of the external pulse [54]. Stable entrainment of NF-κB oscillators (figure 2*f*) requires a minimal duration and minimal concentration of the synchronizing TNF pulse [55]. The sensitivity of fly circadian clock has been tested by varying the duration of light pulses, which mainly affect the degradation of the clock gene *TIM* and can entrain the system in all tissues (figure 2*a*) (both neuronal and non-neuronal tissues in drosophila are photoreceptive) [9,10]. Short light pulses lead to a PRC with a small magnitude and a continuous transition between phase advance and phase delay (called ‘type 1’ resetting) [56] (box 2*b*). As the duration of the light pulse increases, the PRC's magnitude increases (box 2*c*) and may show discontinuity between phase advance and phase delay regions (called ‘type 0’ resetting) (box 2*d*). Around this discontinuity, the new phase after perturbation is highly sensitive to the old phase and may lead to complex behaviours of the system, such as chaos [57,58]. Similarly, the PRC of the circadian clock of cyanobacteria is continuous, i.e. lacks phase singularities, under a short temperature pulse [59]. The phase shift increases with the increase of pulse duration, while the transition between phase advance and delay becomes sharper. Consequently, the PRC exhibits a singularity point above a certain pulse duration. This phase singularity may cause population-level arrhythmicity when certain perturbations cause stochastic phases of oscillations in individual cells. In the case of cardiac pacemaker, discontinuity of PRC has been suggested to lead to cardiac arrhythmias [1,60,61].

Absence of phase shift, i.e. flat curve (box 2*a*), indicates no possibility for entrainment. PRCs with low (box 2*b*) or high (box 2*c*) magnitude on phase shift indicate lower or higher propensity for entrainment. A PRC may have multiple zero points, with well-known examples in the circadian system [48,57,59,62], meaning that a perturbation administrated when the system's phase is at these points will not cause phase change [1]. A PRC may have multiple zeros corresponding to distinct entrainment modes. The slope at a zero point of a PRC dictates the stability of this entrainment state: a negative or positive slope predicts stable or unstable (i.e. further from or closer to uncoupling regions) entrainment, respectively. Last, PRCs can exhibit phase singularities (marked by a vertical line in box 2*d*), at which the phase resetting is very sensitive to the phase at stimulus.

## 6. The impact of entrainment on heterogeneity between individual cells

During entrainment, each single-cell oscillator locks to the external input (figure 1*d*). If the population of cells is initially heterogeneous in its oscillations, phase locking results in a loss of heterogeneity. For example, the glycolytic oscillations of isolated yeast cells (figure 2*e*) display a broad distribution of frequencies around half a minute [63]. Periodic cyanide input can entrain this heterogeneous population through phase shifting (see section above). All cells’ oscillations become synchronized after the first cyanide pulse [12] reducing population heterogeneity. Furthermore, both robust and weak (or non-) oscillating cells entrain to the periodic input, further reducing population heterogeneity.

Entrainment through intercellular communication (figure 1*c*) can also decrease cell-to-cell variability. During glycolysis in yeast cultures, acetaldehyde secreted by cells induces synchronization of metabolic oscillations (even converting non-oscillating cells to an oscillatory state) [64]; this effect occurs only above a minimal cell density [3,19,63,65]. Similarly, dissociated cells of many organs show high heterogeneity of their oscillations. Isolated individual sinoatrial node cardiac pacemaker cells have varying periods [66–69], but at high density, they exhibit the stereotypical 80 beats per minute [61,70]. Dispersed cultures of suprachiasmatic nucleus (SCN) neurons behave as non-synchronous single-cell oscillators and fire with widely varying circadian periods distinct from 24 h [71,72]. When neurons are maintained at high density, either in explants or dispersals, their periods synchronize [5,73] to achieve tissue-level synchrony, in which all cells oscillate at the stereotypical 24 h period. The secreted factor synchronizing circadian oscillations of SCN neurons is less clear than that for glycolysis. Separation of the dorsal and ventral SCN resulted in a loss of synchrony of the neural rhythms of the dorsal (but not ventral) SCN, suggesting that a neurotransmitter released by the ventral SCN maintains synchrony throughout the SCN [74]. Indeed, some of the candidate synchronizing factors (neurotransmitters γ-aminobutyric acid, vasoactive intestinal peptide and gastrin-releasing peptide) changed the firing rate of dorsal SCN neurons [73,75,76].

When an initially heterogeneous cell population entrains through intercellular communication to become more homogeneous, it is not clear which cells will dominate the final behaviour of the population. When two cell suspensions of yeast oscillating out of phase were mixed, synchronization was dominated by the culture whose NADH levels were decreasing [77,78]. A different mechanism operated when non-synchronized oscillating cardiomyocytes were placed into physical contact through a connected agarose microchamber [79,80] to synchronize their beating. The cells synchronized to the one showing smaller fluctuations in beating. Thus, it appears that different mechanisms can be employed to determine which of two functionally equivalent oscillators dominates during entrainment.

In some cases, entrainment can increase population heterogeneity, for example when it involves bi-stable responses. This phenomenon has been observed following periodic stimulation of the NF-κB pathway by the cytokine TNF-alpha (figure 2*f*) [40]. A single pulse of TNF-alpha leads to NF-κB oscillations with a period of 90 min. When the TNF-alpha signal was provided in an oscillatory manner, cells entrained at multiple ratios for a given TNF periodic input. The multi-stability in entrainment ratios depended on the input frequency. When the stimulation period corresponded to the original unaltered period, 90 min, the population entrained nearly homogeneously with a 90 min phase-locked oscillation (1 : 1 ratio). By contrast, during a 150 min stimulation period cells showed a mixture of cellular responses including 150 min oscillation (1 : 1), 75 min oscillation (1 : 2), or without phase locking. Multi-stability rose from extrinsic noise (variation in signalling parameters between cells) that caused a significant broadening of the entrainment Arnold tongues regions (see box 1), revealing an important function of noise in allowing for a heterogeneous response to a periodic stimulus [40].

## 7. Plausible functions of entrainment

Entrainment is a ubiquitous phenomenon in biology, found across species and in diverse systems. In some cases, the function of entertainment is clear. For example, systems in which a population of cells synchronizes to achieve a specific coordinated task, such as the synchronization of SCN neurons to light–dark cycles provides further synchronization in downstream organs [81]. In the cardiac rhythms, synchronization of pacemaker cells provides blood circulation [61]. In systems in which cellular information is encoded in frequency, such as the frequency modulation of the transcription factor Crz1 by extracellular calcium concentration ensuring appropriate downstream expression [82] or frequency of motor protein-based oscillations in neurons is a read-out for axonal length [83]; a potential function for entrainment is to strengthen such a modality of signalling. However, in many other systems, the biological function for entrainment remains unclear. For example, despite its ubiquity, the physiological function of glycolytic oscillations and their entrainment are still uncertain [63]. In addition, while entrainment of NF-κB was shown to coordinate the transcriptional response downstream of NF-κB [40], entrainment in this system was achieved artificially through period stimulation by TNF-alpha, which is not known to oscillate *in vivo*.

Entrainment of biological clocks may also play an important role during evolution. As one example, the oscillation of cyclin-dependent kinase (CDK) activity drives other periodic events, such as DNA replication and chromosome separation, during the cell cycle. Interestingly, CDKs seem to have appeared late during evolution [84], raising the question as to how cells synchronize the series of events required for proliferation prior to CDK emergence. Recent studies in yeast identified several processes that show periodic behaviours even in the absence of CDK oscillator. These CDK-independent oscillators include budding, DNA replication, centrosome duplication, transcription and Cdc14 release [85–89]. Intriguingly, their intrinsic periods are close to the normal cell cycle duration. It has been speculated that cell cycle processes may be intrinsically oscillatory before the emergence of CDK, and these oscillators entrain each other to create an aggregate rhythm [43]. The master CDK oscillator may have evolved to regulate other oscillators in order to yield a stable entrainment structure. This satisfies the evolutionary requirement of utility of intermediate forms [90]. Entrainment of autonomous oscillators could have been important in early cell cycle evolution, raising the possibility that it plays a role in promoting a stable cell cycle rhythm in modern eukaryotes.

## 8. Future perspectives

Many aspects of entrainment remain unexplored mainly due to the complex network interactions controlling and connecting oscillations in biology. One approach that can be useful in disentangling interconnected oscillatory systems is synthetic biology. Synthetic biology allows precise control of entrainment networks and has been used to study extremely complex systems such as a built-in circadian clock [81,91] or quorum sensing [4]. In the future, building synthetic oscillators that are heavily intertwined in nature (such as the cell cycle or the Cdc14 oscillators) could elucidate the mechanisms behind their coupling and avoid the use of genetic manipulation in their original natural systems. Finally, the potential of an oscillatory system to be entrained has not been explored in many networks, even in well studied oscillatory systems such as p53 or Msn2 [29,92,93], both having the potential to be entrained using distinct combinations of drugs.

Advances in technologies such as microfluidic devices, microscopy and optical traps allow precise spatial and temporal control of a cell's environment and facilitate single-cell measurements of oscillatory behaviours. Synthetic biology approaches along with technological advances will be essential to explore fundamental questions of entrainment such as the molecular determinants of the entrainment capability of a system and the functional consequences of entrainment.

## Data Availability

This article has no additional data.
